# Introductory Lectures

**Published:** 1848-05

**Authors:** 


### INTRODUCTORY LECTURES.

In several of our medical schools it is now the practice to have but a single public introductory lecture, but in most the professors adhere to the old custom of delivering a popular discourse prefatory to their several courses. These lectures are not always, nor even generally, introductory to the branches of medicine taught by the professors, but often take the widest range, and treat, if not exactly *de omnibus rebus et quibusdam aliis,* at least of so many things that it would be difficult to say what the author was driving at. Many, no doubt, are published which are written without any view to publication, and of the imperfections of which none can be more sensible than the authors themselves. But it is a question worth considering, what is gained by the publication of lectures so hastily prepared, and felt to be so crude and unfinished? If they reflect no honor upon the authors, it is certain they do no credit to the medical literature of the country. Whether it be to haste or to some other cause that we are to attribute all the defects of these introductory addresses, it cannot be denied that the faults of some are glaring enough. We would indicate, particularly, a floridness of style which is quite too prevalent. One in reading the opening of many of these lectures would fancy that he had got hold of a fourth-of-July oration. The authors seem to contemn plain prose and are never happy except when dealing in tropes and figures. It is time this taste had yielded to one more worthy of the day, and of our profession and country. It is time that we had left this turgid style to sophomores.

Among the numerous introductory lectures published last winter by the classes to which they were addressed, there are not a few which do credit to the taste, judgment, and learning of the authors; but there are also some which in point of style, would justify the utmost severity of criticism. We do not, however, propose indulging in such criticism, for which, at present, we have neither leisure nor inclination.

We need not say that the introductory of Professor Dunglison is free from the sins against which we have attempted to raise our voice. The emanations of his pen are always in good taste. So are the productions of his colleague, Professor Mitchell, whose lecture now before us is one of the best of the season. We cannot say quite so much for the taste displayed by Professor Mutter in his elaborate lecture, but the tone and temper of it cannot be too highly praised.

The lecture of Professor Bedford is the only one we have received from New York. It is what it purports to be—an introductory to his lectures on Midwifery, and one which must have inspired his pupils with higher respect for their profession. Professor Shipman's introductory to his course on Surgery in the Indiana Medical College, is evidently a hasty production and doubtless would have been freed from certain blemishes of manner if the author had taken time to prune and retrench it.

